# Supplementary material for: Causal Relationship between Aging and Anorexia Nervosa: A White-Matter-Microstructure-Mediated Mendelian Randomization Analysis
Source: Biomedicines. 2024 Aug 16;12(8):1874. doi: 10.3390/biomedicines12081874 (PMC11351342; doi:10.3390/biomedicines12081874)
Supplement: Supplementary file 1 [file biomedicines-12-01874-s001.zip › Supplementary Figure/Figure S4/Singe SNP plot of MR analysis for TL to ubm-b-1802.pdf]

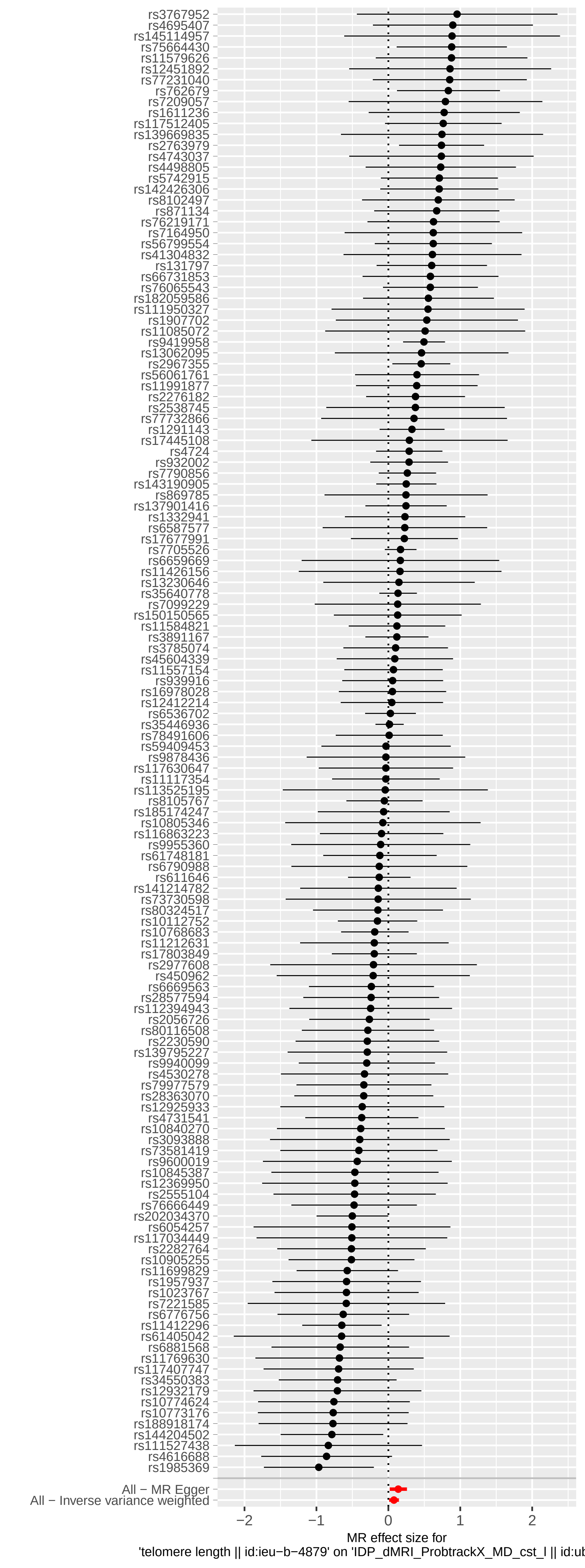
rs3767952
rs4695407
rs145114957
rs75664430
rs11579626
rs12451892
rs77231040
rs762679
rs7209057
rs1611236
rs117512405
rs139669835
rs2763979
rs4743037
rs4498805
rs5742915
rs142426306
rs8102497
rs871134
rs76219171
rs7164950
rs56799554
rs41304832
rs131797
rs66731853
rs76065543
rs182059586
rs111950327
rs1907702
rs11085072
rs9419958
rs13062095
rs2967355
rs56061761
rs11991877
rs2276182
rs2538745
rs77732866
rs1291143
rs17445108
rs4724
rs932002
rs7790856
rs143190905
rs869785
rs137901416
rs1332941
rs6587577
rs17677991
rs7705526
rs6659669
rs11426156
rs13230646
rs35640778
rs7099229
rs150150565
rs11584821
rs3891167
rs3785074
rs45604339
rs11557154
rs939916
rs16978028
rs12412214
rs6536702
rs35446936
rs78491606
rs59409453
rs9878436
rs117630647
rs11117354
rs113525195
rs8105767
rs185174247
rs10805346
rs116863223
rs9955360
rs61748181
rs6790988
rs611646
rs141214782
rs73730598
rs80324517
rs10112752
rs10768683
rs11212631
rs17803849
rs2977608
rs450962
rs6669563
rs28577594
rs112394943
rs2056726
rs80116508
rs2230590
rs139795227
rs9940099
rs4530278
rs79977579
rs28363070
rs12925933
rs4731541
rs10840270
rs3093888
rs73581419
rs9600019
rs10845387
rs12369950
rs2555104
rs76666449
rs202034370
rs6054257
rs117034449
rs2282764
rs10905255
rs11699829
rs1957937
rs1023767
rs7221585
rs6776756
rs11412296
rs61405042
rs6881568
rs11769630
rs117407747
rs34550383
rs12932179
rs10774624
rs10773176
rs188918174
rs144204502
rs111527438
rs4616688
rs1985369
All − MR Egger
All − Inverse variance weighted
−2
−1
0
1
2
MR effect size for
'telomere length || id:ieu−b−4879' on 'IDP_dMRI_ProbtrackX_MD_cst_l || id:ub
